# Efforts to make and apply humanized yeast

**DOI:** 10.1093/bfgp/elv041

**Published:** 2015-10-13

**Authors:** Jon M. Laurent, Jonathan H. Young, Aashiq H. Kachroo, Edward M. Marcotte

**Keywords:** yeast, humanization, evolution, functional genomics, high-throughput assays

## Abstract

Despite a billion years of divergent evolution, the baker’s yeast *Saccharomyces cerevisiae* has long proven to be an invaluable model organism for studying human biology. Given its tractability and ease of genetic manipulation, along with extensive genetic conservation with humans, it is perhaps no surprise that researchers have been able to expand its utility by expressing human proteins in yeast, or by humanizing specific yeast amino acids, proteins or even entire pathways. These methods are increasingly being scaled in throughput, further enabling the detailed investigation of human biology and disease-specific variations of human genes in a simplified model organism.

## Introduction

While direct investigation of human biology can often be limited due to practical or ethical considerations, the wide array of model organisms available to researchers has allowed a remarkable amount of discovery into our own molecular functioning. All living organisms share commonalities in their underlying molecular makeup; thus, knowledge of processes studied in one organism can often be translated to others. Eukaryotic microorganisms, with replication times and tractability akin to bacteria, but much more overlap in cellular components to humans, have proven especially useful in studying human biology. Chief among these, the budding yeast *Saccharomyces cerevisiae* has been an invaluable tool for uncovering much of the basic biology that underlies human cell functioning and disease.

**Figure 1:**
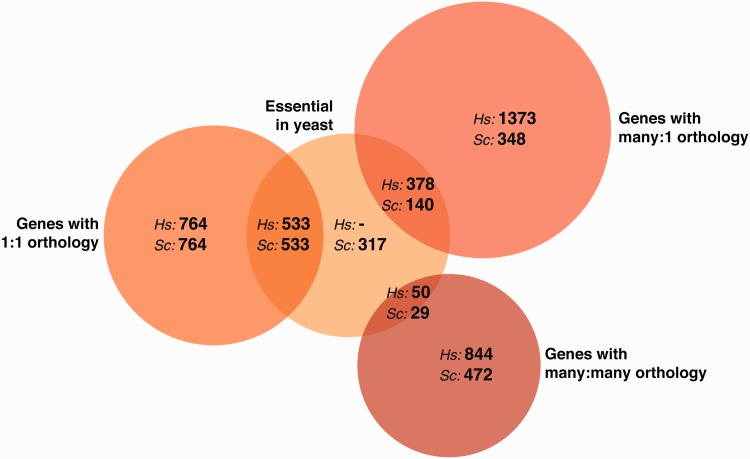
Humans and yeast share thousands of orthologous genes. The Venn diagram illustrates counts of human–yeast orthologs [2], grouped according to the nature of the orthology (classifying orthologs according to whether their count in humans:yeast is 1:1, many:1, or many:many) and whether the yeast genes are essential or not under standard laboratory growth conditions [3]. (A colour version of this figure is available online at: http://bfg.oxfordjournals.org)

**Figure 2:**
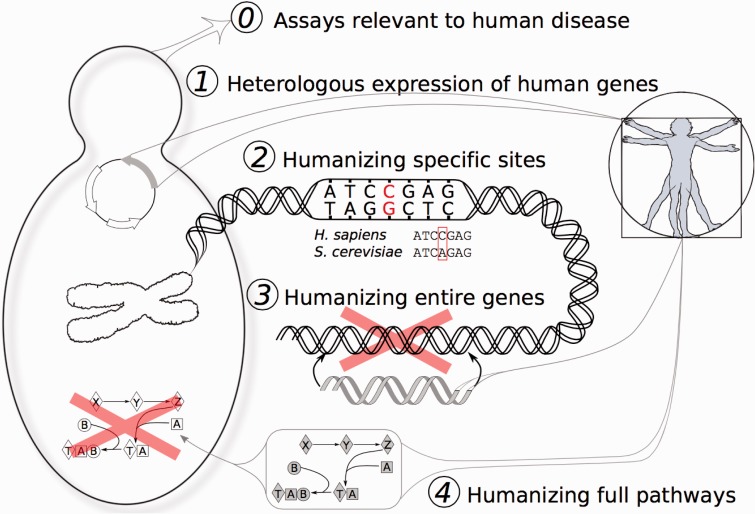
Five degrees of yeast humanization. Yeast have proven useful for the direct study of human biology in a variety of forms, illustrated here to distinguish those cases in which yeast were simply studied for human-specific processes and drugs (degree 0), to the heterologous expression of human genes in yeast (degree 1), all the way to the directed replacement of specific amino acids, genes, and pathways (degrees 2–4, respectively). (A colour version of this figure is available online at: http://bfg.oxfordjournals.org)

The last common ancestor of humans and yeast is estimated to have lived approximately a billion years ago [1], and we still share a substantial portion of our genetic material. The human genome contains roughly 20 000 protein-coding genes while the yeast genome comprises about 6000. A pairwise comparison of genes between the species reveals ∼2100 groups of orthologs, representing 2300 yeast genes and 3900 human genes [2] (Figure 1). Many shared genes perform important cellular roles in both organisms, and their perturbation leads to diverse human disorders, from cancer to Mendelian diseases. The homology between humans and yeast, and the inherent tractability of yeast, has enabled researchers to expand its usefulness as a model for human biology, both by heterologous expression of human proteins, as well as by directly modifying yeast cells to humanize specific amino acids, proteins or even entire yeast pathways (Figure 2).

Two early successes in humanization were demonstrated in 1985 and 1987 as a means of identifying human genes capable of rescuing yeast mutants: First, Kataoka *et al*. [4] expressed chimeric yeast/human or full human *RAS* genes in yeast *Δras* mutants to demonstrate the functional homology retained between the two species. Next, in a famous and elegant experiment to identify human genes possessing the same function as fungal *CDC2*, Lee and Nurse [5] expressed a large library of human cDNAs in fission yeast (*Schizosaccharomyces pombe*), to identify an orthologous protein capable of rescuing a* S. pombe Δcdc2* mutant.

In the 30 years since these experiments, more than 400 yeast genes have been humanized [6–8]. Studies have ranged in their degree of direct translation to humans, from using yeast proteins to identify targets for human drugs to large-scale replacement of yeast genes with their human orthologs [9]. In this review, we discuss these ongoing efforts to develop and utilize humanized yeast, and their increased emphasis on high-throughput construction and applications. We consider five ‘degrees' of humanization, representing increasing levels by which the yeast have been altered to resemble the human case (Figure 2).

## Five degrees of humanization

### Degree 0: Studying nonhumanized yeast to address human biology

While major motivating applications for humanized yeast are in disease gene discovery and drug development, humanizing yeast will also provide a valuable foundation for answering fundamental questions about human biology. The groundwork for the necessary techniques is rooted in studies of nonhumanized yeast to address such questions. Many key genes functioning in conserved cellular processes have been discovered using yeast as a model, including those in such fundamental processes as the cell cycle and DNA replication [10, 11], among others. Fungi also have a long history of use for the discovery of several classes of human therapeutics, perhaps most notably in the development of statin drugs in the 1970s, when the first statins were isolated from fungi [12]. Remarkably, yeast have proven useful not just for the study of human cellular processes, but also processes specific to distinct human tissues and organs, owing to the often surprising evolutionary repurposing of genes between the human and yeast lineages.

Examples of deep homology between human and yeast processes can be clearly seen in the identification of orthologous phenotypes, or ‘phenologs’ [13]. Phenologs are defined as phenotypes that share a significantly overlapping set of underlying orthologous genes. Nonobvious phenologs have been identified between humans and yeast, for example one relating genes involved in mammalian angiogenesis to those involved in the response to lovastatin in yeast [13]. Identification of this particular phenolog ultimately led to the repurposing of a FDA-approved anti-fungal drug, thiabendazole (TBZ), as a promising vascular disrupting agent [14]. Notably, the connection between TBZ and the relevant set of yeast genes was identified on the basis of previously performed high-throughput drug-gene interaction screens [15], a testament to the utility of collecting and consolidating such data for future, often unanticipated, analyses.

In a similar fashion, the obvious parallels between human and yeast mitochondria motivated a screen of the homozygous diploid yeast deletion library to uncover human genes involved in mitochondrial disorders [16]. Steinmetz and colleagues found over half of known mitochondrial genes were required for normal growth on nonfermentable substrates. To discover new mitochondrial genes, they clustered all deletion strains according to their growth on nonfermentable substrates. Over 450 yeast deletion strains exhibited growth defects in these conditions, 255 of which had human orthologs. Of those, 29 were either known to be or were possibly involved in mitochondria-based pathology. Novel candidate disease genes for human mitochondrial diseases were then inferred based on the deletion phenotype of the corresponding yeast ortholog [16].

In the field of drug discovery, yeast provide a useful high-throughput platform both to select candidate drug compounds and to identify drug targets. In perhaps the simplest case, screening an overexpression or deletion yeast strain collection can identify strains that are overly sensitive or resistant to drug treatment. For example, screening a set of kinase-directed compounds against a yeast overexpression library revealed several compounds targeting the *PKC1-MAPK1* pathway. One compound was found to directly target yeast Pkc1 [17]. In a complementary approach, Lum *et al*. [18] assayed a heterozygous yeast deletion mutant library looking for haploinsufficiency in response to a set of known therapeutics and successfully identified protein targets for several compounds.

Other approaches to drug discovery have been made possible by combining chemogenomic screens of the yeast deletion library [15] and high-throughput quantification of yeast genetic interactions [19]. Identifying mutants that are sensitive to a drug of interest relative to wild-type suggests that the drug’s target may be a genetic interaction partner of the deleted gene. Bioinformatic querying of genetic interaction and chemical sensitivity databases has yielded both drug targets and off-target effects for multiple compounds, e.g. tamoxifen and benomyl [20, 21]. Experimental screens of chemical–genetic interactions have also been fruitful. For example, significant genetic interactions between yeast *SOD1* and the DNA damage and checkpoint repair (DDCR) pathway guided the discovery of a small-molecule inhibitor of DDCR in yeast. *Sod1*Δ strains showed sensitivity to several compounds in a screen of over 3000 small molecules. One compound allowed partial rescue of yeast growth inhibition in the presence of DNA-damaging agents, suggesting DDCR as the target for the compound, which was confirmed in human colorectal cancer cell lines [22].

Genetic interaction is also evident when overexpression of one gene inhibits growth in the deletion background of another gene. In some cancers, Mad2, a critical cell-cycle checkpoint control protein, is overexpressed and screening for genes whose deletion causes reduced growth in Mad2-overexpressing yeast identified candidate target genes [23]. Thirteen of the identified yeast genes had human orthologs, and knockdown of one of these (PPP2R1A) caused lethality in human cells (HeLa) that had MAD2 overexpressed. Interestingly, PPP2R1A is a regulatory subunit of protein phosphatase 2 (Ppa2), the target of cantharidin, which was found to inhibit the MAD2-overexpressing osteosarcoma cell line OS-17.

Finally, alternative high-throughput techniques for drug target identification that do not involve genetic interaction screening have also been developed. The molecularly barcoded yeast open reading frame (MoBY-ORF) collection comprises a library of ∼5000 yeast genes cloned in expression plasmids flanked by upstream and downstream barcodes that enable plasmid identification in pooled growth assays, greatly lowering the amount of drug necessary. In the case of a drug-resistant mutant, the MoBY-ORF collection is transformed into mutant strains and assayed for renewed sensitivity to the drug, followed by amplification and identification of the responsible gene via microarray [24]. The method was validated by identifying targets of rapamycin and several antifungals.

### Degree 1: Expressing human proteins in yeast regardless of orthology

To expand on the utility of screening yeast libraries for disease-causing proteins and possible drug targets, researchers have taken advantage of the ability to easily transform yeast with exogenous DNA. Heterologous over-expression of human proteins, without regard to any orthology, can cause phenotypic changes in yeast that can then be screened against. One study expressed ∼50% of all human cDNAs in yeast and found that approximately 6% caused toxicity [25]. Moreover, about 25% of the toxic cDNAs had unknown functions. Other studies have chosen to express human genes in yeast that are associated with human disease, such as cancer and neurological disorders. In this section, we’ll explore a few noteworthy examples.

One such case involves the poly ADP ribose polymerase (PARP) family of proteins, studied as a therapeutic target for breast and ovarian cancers [26]. Notably, yeast has no PARP homolog, so efforts to use yeast as a platform for discovery of PARP inhibitors necessitated heterologous expression of human PARP. As expression of PARP1 or PARP2 leads to yeast growth inhibition, small-molecule compounds that rescue growth should point to candidate PARP inhibitors. Screening of 16 000 organic small-molecules led to the discovery of two compounds that effectively rescued growth of yeast expressing human PARP1 and PARP2 [27]. Coexpression of a second human protein, PARG, alongside PARP1 or PARP2 also restored yeast growth to nearly wild-type levels. Such screens illustrate the utility of human drug screens in yeast, here taking advantage of the fact that yeast lacks the relevant system.

**Figure 3:**
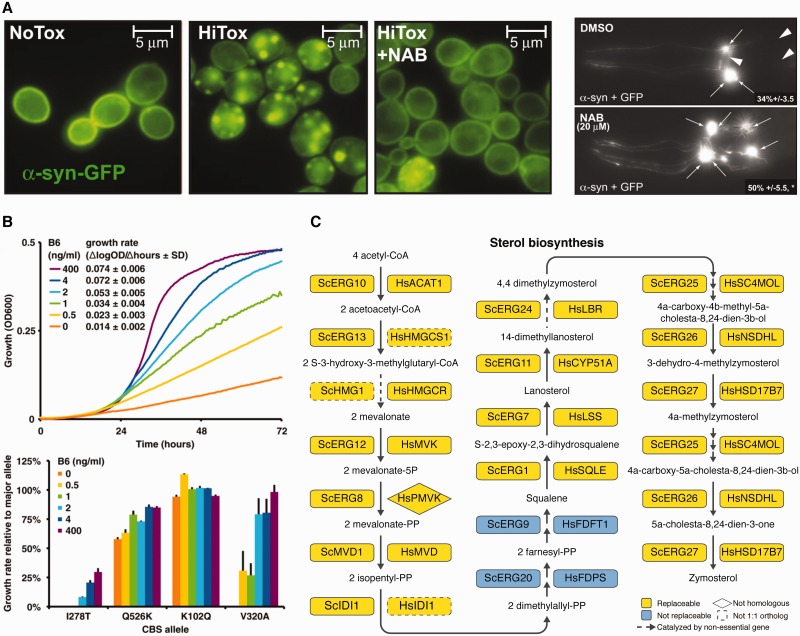
Three examples of humanized yeast, relevant to neurodegenerative disorders, metabolic disorders and cholesterol biosynthesis. **(A)** (Left) Yeast show diffuse distribution of α-synuclein during moderate expression (NoTox), aggregation during overexpression (HiTox) and rescue of aggregation by administration of an N-aryl benzimidazole (NAB). (Right) Degeneration (white arrowheads) of *C. elegans* DA neurons during overexpression of α-synuclein (top) and protection by NAB administration (bottom). Figures are adapted from Tardiff *et al*. [30] with permission. **(B)** (top) Growth over time of *Δcys4* yeast expressing the major human allele of cystathione-β-synthase (*CBS)* as a function of varying concentrations of vitamin B6. (bottom) Growth rate at various levels of vitamin B6 for several minor human alleles, relative to the major human allele. Figures are adapted from Mayfield *et al*. [31] with permission. **(C) **Seventeen of 19 tested genes of the yeast sterol biosynthesis pathway are replaceable by their human equivalents. Figure adapted from Kachroo *et al*. [6] with permission. (A colour version of this figure is available online at: http://bfg.oxfordjournals.org)

Yeast has proven a useful platform even for neurological disorders. For example, the human proteins TDP-43 and FUS are critical for amyotrophic lateral sclerosis (ALS), but there may be other important genes involved in related disorders. In one study, yeast was used to search for additional candidate disease genes for ALS. As both TDP-43 and FUS contain RNA recognition motifs (RRM), a functional screen was designed in which 133 human open reading frames (ORFs) that also contain RRMs were inserted into plasmids and tagged with YFP under a galactose-inducible promoter. Screening for human ORFs that mimicked the formation of cytoplasmic foci by TDP-43 and FUS identified 38 candidate human ORFs. Bioinformatic and experimental validation in other models strongly implicated *TAF15* as a novel candidate ALS gene [28]. Further screening of approximately 200 000 compounds revealed several 8-hydroxyquinolines that rescued human Tdp-43-induced toxicity in yeast [29]. An *N*-aryl benzimidazole (NAB) compound from the same screen was identified as having a greater protective effect from α-synuclein toxicity in a yeast model of Parkinson’s disease (Figure 3A) [30]. A screen against several NAB analogs revealed that more effective rescue of α-synuclein toxicity predicted greater growth inhibition of wild-type yeast, suggesting that α-synuclein rescue and growth inhibition had related mechanisms of action. This observation motivated a follow-up screen against a yeast mutant library to identify mutants allowing growth in high concentrations of NAB2, a NAB analog. The screen revealed yeast protein Rsp5 (human ortholog NEDD4) as the target of NAB2, which was further shown to rescue α-synuclein toxicity in Parkinson patient-derived neurons [32].

Yeast has also been used as a model to investigate how Alzheimer’s disease (AD) risk factors relate mechanistically to amyloid beta (Aβ) peptide toxicity. After establishing that expression of human Aβ in yeast caused toxicity, Treusch and colleagues [33] screened >5500 yeast ORFs to identify those whose over-expression suppressed or enhanced Aβ toxicity. Overexpression of these genes’ worm or human homologs, notably the gene *PICALM*, confirmed their ability to abrogate Aβ toxicity in *C. elegans* and rat cortical neurons. Further investigation demonstrated that, at least in yeast, Aβ toxicity occurred by disrupting endocytic trafficking.

Expressing nonorthologous human proteins in yeast has thus yielded several promising results and potential targets for therapy. These techniques are limited by the fact that not all human proteins, when overexpressed, will have an assayable phenotype, especially one as straightforward as growth inhibition or rescue. However, the extensive conservation of genes between humans and yeast (Figure 1) opens up many potentially important avenues to explore using humanized yeast, as we discuss next.

### Degree 2: Humanizing specific positions within native yeast genes

Based on aligned amino acid sequences of orthologous yeast and human proteins, a number of researchers have opted to mutate native yeast proteins to match the human sequences at key positions. In particular, evolutionarily conserved residues are often important for protein function, and show an elevated frequency of being involved in human disease [34]. If a mutation in a specific residue is associated with a disease phenotype in humans, a similar substitution in its yeast equivalent may reveal its effect on the protein and at the organism level. This notion has led to the investigation of many yeast proteins while making substitutions in the context of their human counterparts.

One area in which this site-specific humanization has been particularly fruitful is in the study of mitochondrial function, whose disruption causes diverse diseases [35]. Yeast have strong experimental advantages here, including a high degree of conservation between human and yeast mitochondria, and yeast’s ability to grow in the absence of functional mitochondria with a ‘petite’ phenotype. One such abnormality is caused by mutations in the yeast *MIP1* gene, which encodes DNA polymerase gamma. Mutations in *POLG*, the human ortholog of *MIP1*, have been implicated in several human diseases [36], and in agreement with this trend, Stuart *et al*. [37] observed a range of yeast phenotypes involving altered proofreading ability and rates of mtDNA replication, after having made human disease relevant site-specific mutations at equivalent positions in the yeast protein. A follow-up study identified conditions that rescued yeast *MIP1* mutant phenotypes analogous to human progressive external ophthalmoplegia-associated *POLG* mutations [38].

Along with DNA replication, precise translation of the encoded information is important for optimal mitochondrial function. Mto1 is a protein involved in translational fidelity in both humans and yeast by catalyzing the 5’-carboxymethylaminomethylation of the wobble uridine in three mitochondrial tRNAs. Mutations in human *MTO1* have been implicated in hypertrophic cardiomyopathy and lactic acidosis [39]; introduction of the corresponding mutations into yeast *MTO1* result in a defective enzyme, which only partially rescued a Δ*mto1* yeast mutant [39].

Beyond the study of mitochondria, humanized yeast have also been useful for the study of nuclear DNA maintenance, an ancient and highly conserved set of processes. One conserved gene, *MSH2*, recognizes mispaired bases in DNA. Mutations in human *MSH2* have been implicated in hereditary nonpolyposis colorectal cancer. Gammie *et al*. [40] assayed 54 known disease mutations in *MSH2* by engineering them into the analogous positions in the yeast gene, and found that over half (55%) displayed strong defects in mismatch repair assays. Roughly half of the mutations resulted in decreased Msh2 protein levels or disrupted important interactions.

Proper translation of nuclear encoded genes involves addition and recognition of the poly-A signal typical of cellular mRNAs. *PAB1* encodes a poly-A binding protein important for interfacing with translation via binding with eIF4G1/2. Melamed *et al*. [41] took advantage of recent developments in deep mutational scanning to substitute 197 amino acid changes from 52 Pab1 homologs into the yeast protein. Three of 17 deleterious mutations corresponded to the human residues, and a yeast mutant with all three of the human residues enabled yeast Pab1 to switch its binding specificity from yeast eIFG1 to the human ortholog.

A recent exome sequencing effort directed at 67 T-cell lymphoblastic leukemia patients identified several new oncogenic driver genes [42]. Two of these, *RPL5* and *RPL10*, are involved in ribosome biogenesis. A recurrent T-ALL *RPL10* mutation, Arg98Ser, occurs in a conserved residue and was introduced into yeast *RPL10* and the yeast were observed to have defects in ribosome biogenesis. Detailed investigation of the Arg98Ser mutant in yeast revealed that it allowed bypassing of late 60S subunit maturation quality control, but could be genetically suppressed by secondary mutations, possibly leading to oncogenesis [43]. These studies highlight yeast as a useful simplified model for deciphering disease mechanisms.

While these studies offer some insight into the utility of humanizing specific residues, there are theoretical caveats to the method. Although important functional residues may be conserved, they have evolved within the context of their extant protein sequence, and as such the functional consequences of many human-specific mutations may be abrogated or ‘hidden’ in the context of the yeast protein. Additionally, many disease-associated residues are not conserved, and in fact many human disease alleles are fixed in other species [44]. Thus, analogous residues in yeast may not be functionally equivalent [45]. Such caveats have led researchers to instead humanize entire genes, rather than amino acid positions.

### Degree 3: Humanizing entire yeast genes

One route around the limitations of humanizing specific amino acids is to functionally replace an entire yeast gene with a human counterpart. Such studies are enabled by the ease with which the yeast genome can be manipulated and exogenous proteins can be expressed. The last 30 years have seen numerous cases of such humanization, first used as a method for identifying functionally orthologous human proteins well before the genomes of human or any fungi were sequenced [4, 5].

In an elegant strategy for identifying important human SNPs, Marini *et al*. [46] introduced a *FOL3* deletion into a *Δmet13* yeast strain expressing human methylenetetrahydrofolate reductase (MTHFR). This allowed them to titrate intracellular folate levels, which are known to have an effect on MTHFR function. They then sequenced the *MTHFR* locus from 564 human individuals and identified 14 nonsynonymous alleles. Assays of these alleles in yeast growth assays revealed that five variants affected the ability of the human protein to complement the yeast mutant, four of which could be abrogated by folate supplementation. The results suggest that even common alleles can have significant effect on protein function.

A related study tested the function of another important metabolic enzyme, cystathione-β-synthase (CBS). CBS deficiency in humans has several severe symptoms, including homocystinuria and mental retardation, among others. Human *CBS* was previously shown to complement deletion of its yeast ortholog, *CYS4* [47]. Eighty-four *CBS* alleles from human patients with homocystinuria were used to replace yeast *CYS4*, and assays for growth and enzyme function revealed that 71 were easily distinguishable from the major human allele and 32 alleles could be rescued with supplementation with one of two CBS cofactors, vitamin B6 or heme (Figure 3B) [31].

Early attempts to humanize yeast also focused on telomerase, implicated in many human cancers and considered a potential target for cancer therapy [48]. Initial approaches to humanizing telomerase included replacing the entire yeast telomerase template by the human version [49]. The resulting yeast strains were viable with normal growth, and Southern hybridization verified that human telomeric sequences (TTAGGG)*_n_* were added to the ends of yeast chromosomes. Subsequent work attempted to replace the yeast catalytic telomerase subunit with the human version. However, despite successful reconstitution of both the human catalytic subunit and template in yeast, no polymerization by human telomerase occurred at the yeast telomeres [50]. Even with the inability of human telomerase to complement its yeast counterpart, recent work demonstrated the utility of expressing human telomerase in yeast for drug screening. A fusion protein of yeast Cdc13 and human telomerase (Cdc13-hTERT-FLAG) was found to cause growth arrest when coexpressed with the human telomerase template [51]. Screening of a 678 compound library found three small-molecules that rescued growth and inhibited human telomerase *in vitro*.

Such replacements need not be limited to orthologs. In a recent study related to the *POLG* mutations described in the previous section, researchers expressed full human POL-γ in a *Δmip1* yeast strain while retaining the yeast Mitochondrial Localization Signal. The slow growth and petite phenotype of the knockout was partially rescued by expression of the orthologous human polymerase subunit POL-γA, but full rescue was observed only when POL-γA was coexpressed with the human accessory subunit POL-γB, which has no known ortholog in yeast. Further characterization by expressing disease-associated human *POLG* variants and comparing them to the wild-type revealed correlations between polymerase fidelity and progression of disease in humans [52], in some cases linking the disease phenotype to catalytic defects in human enzyme [53].

While there have been several published cases of individual human genes complementing yeast mutants, it is unclear how widespread the phenomenon is. One early investigation found that of 25 tested human genes, six were able to complement the yeast mutants [54]. To systematically address this question, a large-scale study from our group recently showed that functional replaceability is a property of many genes (200 of 424 tested) [6]. This general replaceability was surprisingly not primarily driven by the sequence similarity of the two proteins. Rather, genes in particular pathways or complexes tended to be similarly replaceable or not, such as proteasome genes and sterol biosynthesis genes (Figure 3C) being nearly entirely replaceable. Further characterization of proteins in these processes showed that at least one noncomplementing human protein, the proteasome subunit β2, could be made to replace the yeast gene following a single amino acid substitution, and also revealed another case of nonorthologous functional replacement, that of human *PMVK* replacing yeast *ERG8. *Similar to the CBS study described above, our study showed that common alleles of human mevalonate kinase (*MVK*) could be distinguished from disease-associated alleles through simple growth assays in yeast *Δerg12* mutants [6].

Of course, replacing yeast genes with those from humans is not without its own caveats. Analogous to specific humanized sites lacking the context of their human protein, fully humanized proteins may be limited in their ability to interact with their proper partners in the context of a yeast cell. Indeed, the modular nature of replaceability suggests that this may be the case, and hints that inability to properly form necessary interactions is a driving force behind certain proteins not being able to replace their yeast counterparts.

### Degree 4: Humanization of full pathways and complexes

Given the degree to which yeast proteins can be successfully humanized one by one, and the modularity that governs successful individual replaceability [6], an obvious extension of the phenomenon is to humanize entire pathways or complexes. This method mitigates the issue of single gene replacement not capturing the entire functional context of a protein. It should also allow for studying more complicated phenotypes involving multiple members of a pathway or epistatic interactions between multiple genes. Of course, successful humanization of a pathway or protein complex requires that its output also be functionally interchangeable with its yeast counterpart to ensure viability of the cell, and likely requires that corresponding yeast pathways are sufficiently disabled.

As of this writing, few attempts to humanize entire pathways have been carried out. However, one study in particular is worth noting: While this study did not employ true humanization of yeast genes, it nonetheless imparted the ability to glycosylate proteins in a manner highly similar to that of humans. Yeast provides a good platform for high-yield recombinant enzyme production for industrial purposes, but is hindered by the fact that fungi typically produce a high-mannose glycosylation pattern highly dissimilar to the more complex and heterogeneous glycosylation pattern typical of human proteins [55]. A key first step in the implementation of this pathway was removal of yeast mannosylation enzymes in order to eliminate any interference from the native pathways. The researchers then used a high-throughput strategy to fuse fungal golgi and endoplasmic reticulum localization domains to catalytic mannosidase and glycosyltransferase domains from across the tree of life to create functional and properly localized glycosylation enzymes expressed in *Pichia pastoris*, an important industrial yeast species [56]. The researchers were able to combine this strategy with heterologous expression of nucleotide sugar transporters and human and mouse sialylation enzymes to express human proteins with correct human glycosylation and sialylation patterns [57, 58], forming a key platform technology for the biotechnology company GlycoFi.

A major limitation of the extent to which pathways may be humanized is the amount of knowledge of required pathway members. This is illustrated in a recent attempt to introduce the human selenocysteine machinery into yeast, which have lost the ability to synthesize and incorporate selenocysteine into proteins. The group developed a method for sequential genomic insertion of multiple exogenous genes and used it to introduce and express 11 human proteins either known or implicated in selenocysteine biosynthesis or incorporation. The use of a selenoprotein reporter revealed that the engineered pathway was not able to incorporate selenocysteine, though it is unclear if this was due to missing factors or interference from yeast components [59]. Despite failure of the pathway to produce a functional output, this remains the most significant whole pathway humanization effort published to date.

While no large protein complexes have been humanized to date, there have been a number of reported cases of small (two subunit) protein complexes being successfully humanized. One case mentioned previously saw full growth rescue of a yeast *mip1Δ* strain only when both subunits of the human POLG complex were expressed simultaneously. In an additional case, simultaneous expression of human proteins CDC7 and DBF4, together forming the DBF4-dependent kinase (DDK) complex, was able to rescue *Δcdc7* or *Δdbf4* yeast strains, while expression of either single human protein could not [60]. Following the same pattern, human ALG13 and ALG14 when expressed together could rescue knockout of either yeast ortholog but neither human protein could do so alone [61]. Expression of both subunits of the human tRNA m^1^A58 methyltransferase was able to rescue knockout of the yeast proteins, and further investigation showed that the methyltransferase specificity and function was retained in the context of the yeast [62].

### Future outlook

While researchers have been humanizing yeast for at least the past 30 years, the application of high-throughput and large-scale yeast technologies to the field is relatively new. Nearly one-fifth of our genes are represented in budding yeast, yet only about one-fifth of that set have been tested for their ability to functionally replace yeast genes. The high rate of complementation seen from efforts to date underlines the need to complete testing of the available orthologs. Many of the remaining genes have lineage-specific duplications that will likely require unique assay methods, but provide ample opportunity to study many human disorders and answer deep evolutionary questions.

Importantly, we need not restrict ourselves to replacing only orthologous genes. While likely not a widespread phenomenon, complementation of yeast mutants by nonorthologous human genes may prove useful, especially in cases where orthologs do not complement or where orthologs can not be identified, such as the functional replacement of yeast *ERG8* by the nonorthologous human *PMVK* gene (Figure 3C) [6]. Given large available libraries of human ORFs [63] and yeast mutant strain collections [3, 64, 65], such studies can potentially be carried out in a high-throughput manner.

Although current examples are limited, humanization of whole pathways will surely be an area of exciting research moving forward. Recent studies suggest it is highly feasible that entire processes can be humanized [6], provided technical hurdles can be successfully overcome [66–68]. Yeast strains with humanized systems and pathways offer great potential as platforms for studying human disease, especially polygenic diseases that arise from the interaction of many alleles across multiple genes, whose manipulation and assay could be greatly simplified in yeast.

Perhaps the most exciting promise for humanized yeast is the potential for constructing ‘personalized' strains, expressing any given allele of a human gene, or combinations of genes, to make personalized yeast ‘avatars'. One can envision a rapid pipeline to identify critical mutations responsible for a patient’s disorder, expressing the relevant alleles in yeast and screening the yeast against available treatment options to identify which therapies may be best. Strains harboring partly or fully humanized pathways or genetically interacting groups of genes will further lend themselves to assaying combination therapies and are natural systems for identifying important human variants by deep scanning mutagenesis [69].

Finally, with the ever expanding body of knowledge regarding functional complementation, it is necessary that results of such experiments be consolidated so interested researchers can easily access them. Notably, the Saccharomyces Genome Database curators have recently begun including human complementation data in their YeastMine database tool (accessible at http://yeastmine.yeastgenome.org) [8]. The Princeton Protein Orthology Database also provides a curated database of cross-species complementation results for several model organisms [7]. Inclusion of these results in human gene databases, such as UniProt and Ensembl, should be a future priority, as should distribution of humanized strain collections.

Key Points

- Despite being highly divergent, yeast remains a valuable model organism for the study of human biology and disease.
- A large proportion of yeast protein-coding genes that have been tested can be replaced with their human orthologs.
- Construction of yeast strains with humanized genes and pathways, combined with already developed high-throughput techniques in yeast research, opens up many possibilities for study of human genes and processes in a simpler organismal context.
